# Effect of α-Amylase on the Structure of Chia Seed Mucilage

**DOI:** 10.3390/biomimetics7040141

**Published:** 2022-09-23

**Authors:** Francesco Piazza, Matilde Colella, Giuseppe Cinelli, Francesco Lopez, Ivan Donati, Pasquale Sacco

**Affiliations:** 1 Department of Life Sciences, University of Trieste, Via Licio Giorgieri 5, I-34127 Trieste, Italy; 2 Department of Biosciences, Biotechnology and Biopharmaceutics, University of Bari “Aldo Moro”, Via Orabona 4, I-70126 Bari, Italy; 3 Department of Agricultural, Environmental and Food Sciences (DiAAA) and Center for Colloid and Surface Science (CSGI), University of Molise, Via De Sanctis, I-86100 Campobasso, Italy; 4 AREA Science Park, Loc. Padriciano 99, I-34149 Trieste, Italy

**Keywords:** chia mucilage, α-amylase, rheology, structural studies, thickeners, food

## Abstract

Thanks to its nutritional and mechanical properties, chia seed mucilage is becoming increasingly popular in the food industry as a small biomolecule. The mechanical properties of an ingredient are a key element for food appreciation during chewing. Therefore, with this study, we explore for the first time the structural changes that chia seed mucilage undergoes when treated with α-amylase, the most abundant enzyme in human saliva. First, rheological time-sweep tests were performed on samples with different enzyme and constant chia mucilage concentrations. Then, the effect of increasing the chia mucilage concentration at a constant enzyme content was investigated. The results show that structural changes occur after enzyme treatment. Rheological measurements show a thickening of the material with an increase in the elastic modulus depending on the concentrations of α-amylase and chia used. This effect is attributed to the release and aggregation of insoluble fibrous aggregates that naturally form the mucilage after the cleavage of the α-1,4-glucoside bond between the α-D-glucopyranose residue and the second β-D-xylopyranose residue by α-amylase. Thus, our data suggest an α-amylase-mediated restructuring of the chia mucilage network that could have implications for the commercial processing of this material.

## 1. Introduction

Hydrogels are water-swollen, cross-linked polymers characterized by covalent bonds or physical interactions that give such matrices their particular mechanical properties and are thus essential for modulating their ultimate application [1,2,3,4,5,6]. Hydrocolloids are suspensions of hydrophilic polymers capable of forming viscous solutions or assembling hydrogels when dispersed in water. In addition to their applications in the biomedical field [1,7,8], these materials have also attracted the interest of the food industry because of their ability to alter the rheological behavior of ingredients, particularly viscosity and texture, which are essential for food appreciation [9,10,11,12]. In such systems, the specific molecular interactions between the polymers that comprise them play a fundamental role [11,13]. Therefore, hydrocolloids are valuable food additives used as thickeners, gelling agents, or emulsifiers, to name just a few of their many applications [14].

Thickening of food is a practice usually used to ensure safe ingestion and to avoid swallowing difficulties, as it slows down the rate of movement of the liquid. Several of the currently available thickeners are starch-based hydrocolloids, such as modified starch, a common and inexpensive thickener that does not alter the taste of foods when used at low concentrations [9]. However, as this product is only moderately hydrolyzed in the mouth by salivary α-amylase, it reduces the viscosity of samples.

Among the natural hydrocolloids used in the food industry (such as starch, pectin, agar, alginate, xanthan gum, guar gum, etc.), chia seeds extracted from *Salvia hispanica* have recently gained wide acceptance thanks to their excellent nutritional value and their ability to release chia seed mucilage, a polysaccharide mucilage produced from the seed coat during hydration [15,16].

Chia mucilage (CM) is an interesting and promising additive for the production of innovative materials [17]. Recently, this mucilage has been studied in detail from a structural point of view [18,19,20]. CM consists of about 60–75% crude fibers and 35% carbohydrates [19,21,22]. The polysaccharide milieu is structurally composed of a repeating tetra-saccharide unit consisting of (1→4)-β-D-xylopyranose-(1→4)-α-D-glucopyranose-(1→4)-β-D-xylopyranose units with recurrent branches at the O-2 of the β-D-xylopyranose residue [14,23]. The great potential of chia seed mucilage is based on several aspects. The ability to bind water and form hydrogels makes CM an excellent thickening agent in foods. Due to its rheological properties, it has been suggested as a fat substitute in various foods [24]. In addition, it has promising biological and therapeutic applications due to its biocompatibility and non-toxicity [19]. Recently, a study has been reported on the in-depth structural characterization of chia mucilage for food processing based on physical ageing [20].

α-Amylase is an enzyme that hydrolyses the α-1,4-glucoside bonds of starch to form maltose, which in turn is cleaved into two glucose molecules by maltase [25,26]. In the human body, α-amylase is produced mainly by the salivary glands and pancreas, but it is also expressed by cells in other tissues, such as the epithelial cells of the small intestine, where it has been shown to be important for cell proliferation and differentiation [27,28]. In vegetables, α-amylase is found mainly in the reserve tissues of vegetables during the period of starch mobilization. The role of α-amylase in the degradation of storage starch in the endosperm of germinating cereal seeds and in the degradation of transitory starch in chloroplasts is well studied [29]. It has already been demonstrated that the main polymer backbone, together with the side branches, is severed by α-amylase, which exerts a versatile effect on the biodegradation of low-density polyethylene in polymer–starch mixtures [30]. Significant differences in the susceptibility of the polysaccharide starch to amylolysis have been reported in different botanical species [31]. The type of amylase and the changes that occur in the starch structure during hydrolysis could thus significantly affect the properties of an ingredient and are therefore of great importance in household and commercial food processing.

As the use of chia mucilage in foods increases, it is important to investigate what changes this ingredient undergoes during its digestion by the α-amylase produced by the human body. It is well known that the perception of texture and viscosity in the mouth plays a key role in food evaluation and preference. Therefore, the change in rheological properties after oral processing of foods is nutritionally important [10,12].

Oral processing of foods involves many tasks, such as the mechanical chewing of solid materials and the enzymatic cleavage of polysaccharides into sugars. The latter occurs thanks to the mixing of food with saliva containing α-amylase [28]. It has been demonstrated that the enzymatic cleavage of polysaccharide chains during the amylolytic pre-digestion process in the mouth leads to consistent changes in the mechanical properties of the manipulated material, especially a significant reduction in viscosity [28,32].

The modification of polymers is a well-defined phenomenon and a new organization of the polymer chain can affect the specific application. In fact, once formed, the gel structure is not necessarily static and can be influenced by a number of dynamic parameters that can lead to gel instability. For this reason, hydrogels that are altered by various factors such as ageing, temperature, and impurities should be carefully considered when developing a product to be used in different applications. For example, one of the behaviors observed in this type of matrix was related to a change in viscosity after storage [33]. Recently, the importance of the structural change induced by ageing phenomena has been demonstrated for chia seed mucilage [20].

In light of these considerations, in this work we provide new insight into the enzyme-induced changes in chia structure. To achieve this goal, we mixed chia seed mucilage and α-amylase at different concentrations and investigated the mechanical changes that occur in the mixtures over time by rheological analysis. These data, together with the studies reported on starch, could indeed provide useful information not only for the food industry but also for human health issues.

## 2. Materials and Methods

### 2.1. Materials

Chia seeds (Salba grain organic) were obtained from I.P.A. s.r.l. Industria Prodotti Agroalimentari (Viterbo, Italy). Phosphate buffer saline (PBS) and α-amylase (α-amylase from *Bacillus* sp., code 10070) were purchased from Sigma-Aldrich.

### 2.2. Chia Mucilage Preparation

Chia seeds were weighted and placed in a vessel with ultrapure water in a 1:20 (*w*/*w*) ratio for 12 h to extract the chia mucilage (CM). The CM was recovered through vacuum filtration with a Buchner funnel, frozen at −40 °C and then freeze-dried under vacuum in a Genesis 25 ES (VirTis, Stone Ridge, NY, USA) for 48 h (maximum shelf temperature 20 °C). After freeze-drying, the CM had the appearance of a dried foam and was stored at room temperature [20]. Then, freeze-dried chia mucilage was dissolved in phosphate buffer (10 mM, pH = 7.4) at *T* = 50 °C with vigorous stirring overnight. CM suspensions were analyzed immediately after preparation. The concentrations used (0.7%, 0.8%, and 1% *w*/*v*) were obtained by serial dilutions. This concentration range was chosen because it represents the optimal conditions with which to operate. Lower concentrations led to the loss of the gel behavior; higher ones led to heterogeneous gel-like networks that made measurements difficult. Considering the heterogeneity of chia mucilage when dissolved in water, it is important to note that the chia suspensions (which came from the same polymer batch) were prepared immediately before the different experiments, in this study.

### 2.3. α-Amylase/Chia Mucilage Mixture Preparation

Amylase tests were performed in the presence of different concentrations of α-amylase (1.3 mg/mL, 2.6 mg/mL, 5 mg/mL, 15 mg/mL, and 20 mg/mL) solubilized in phosphate buffer (10 mM, pH = 7.4). In rheological tests, 100 µL of α-amylase were added to 400 µL of CM suspension for each sample. The total 500 µL of solution were mechanically mixed and quickly loaded onto the rheometer stage. For the qualitative experiments, the same chia–amylase ratio was maintained for a total sample volume of 7 mL.

### 2.4. Qualitative Test

Three solutions were prepared: 7 mL chia mucilage 1% *w*/*v*, 7 mL chia mucilage 1% *w*/*v* + α-amylase 1.3 mg/mL, and 7 mL chia mucilage 1% *w*/*v* + α-amylase 2.6 mg/mL. These solutions were stirred at room temperature and monitored over time. After a set time of 1 h, the solutions were allowed to rest for 5 min, then a photo was taken of each solution, and finally they were placed again under stirring. This step was repeated over 12 h.

### 2.5. Rheological Measurements, Time Sweep Experiments

Rheological measurements were performed using a HAAKE MARS III rheometer (Thermo Scientific, Waltham, MA, USA). To characterize CM suspensions a cone-plate PP35 geometry was used as upper plate and a PP60 as lower plate. Rheology time sweep experiments were carried out under strain-controlled conditions, keeping a deformation γ of 0.1% constant throughout the experiment; frequency, υ, was set at 1 Hz, time at 600 s, and temperature at 37 °C. As the first step, chia mucilage 1% *w/v* mixed with different α-amylase concentrations (2.6–20 mg/mL) was tested. Then, α-amylase concentration was kept fixed at 2.6 mg/mL and chia mucilage ranging from 0.7% to 1% *w*/*v* was used. Storage modulus (G’) data analysis was performed by normalization, subtracting the values obtained from chia seed mucilage without the addition of the enzyme.

## 3. Results

### 3.1. Role of α-Amylase in Polymer Degradation

**Figure 1:**
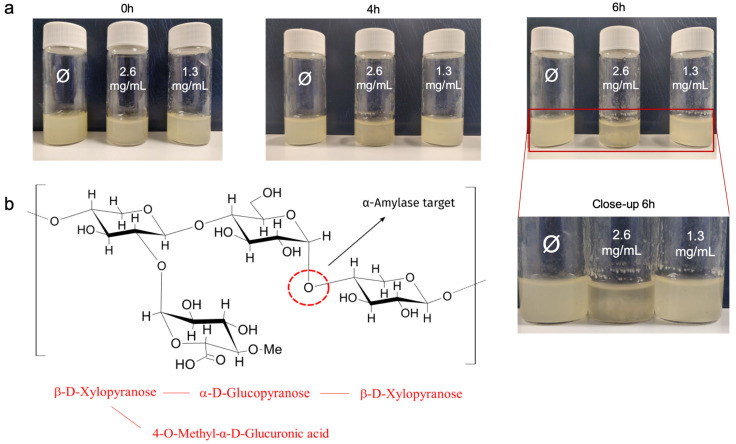
Images of chia suspensions with different α-amylase concentrations over time (0–6 h) with a close-up of the samples at 6 h to better see the differences (**a**), and illustration of the repeating unit of the chia mucilage tetrasaccharide and the putative cleavage site of the α-amylase (**b**).

A previous study by our group highlighted the changes in the structural integrity of the chia mucilage network caused by physical ageing through a full chemical and mechanical characterization [20]. Analysis of FTIR spectra showed that the number of glycosidic bonds decreased after a few days, and images from SEM confirmed the deterioration of the network over time. Therefore, an additional step was to investigate the structural change induced by enzymatic activity. α-Amylase is an endo-acting enzyme that hydrolyses the α-1,4-glycoside bond of polysaccharides by a progressive pattern of action [34]. In plants and animals, it plays the major role in the breakdown of starch reserves or starchy foods into shorter oligosaccharides, mainly maltose [26,29,35,36]. The highly branched tetrasaccharide of chia mucilage has an α-1,4-glucoside bond between its α-D-glucopyranose residue and the second β-D-xylopyranose residue, which is expected to be cleaved by α-amylase (Figure 1b). As with any enzyme catalysis, the hydrolytic process is influenced by environmental conditions such as pH and temperature, as well as enzyme concentration. Furthermore, the efficiency of enzymatic starch degradation is slowed down when the substrate has a solid crystalline structure, as is the case with the solid starch granule [26].

Initially, we decided to qualitatively investigate the polymer degradation triggered by the enzyme within 12 h at pH 7.4 and room temperature. Figure 1a shows images of the chia mucilage (CM) solutions in the presence of different α-amylase concentrations (0–2.6 mg/mL) at different incubation times (0–6 h). This time period was chosen because there were no visible changes after 6 h of incubation. α-Amylase at a concentration of 2.6 mg/mL was used, as this is the average amount of *α-*amylase in human saliva [28]. From this inspection, it appears that the structural changes in CM induced by α-amylase were obviously dependent on the enzyme concentration. We can assume that α-amylase is able to break down the repetitive tetrasaccharide, producing oligosaccharides that are more available for further digestion in food applications and digestive processes. This result is consistent with the behavior observed for starch [26,31]. A brief analysis of the degradation kinetics shows that despite the fact that the process takes place in a short period of time, the difference observed when the enzyme concentration is increased is probably due to the higher accessibility of the specific sites of the chia polysaccharide to the enzyme (*vide infra*).

### 3.2. Impact of Amylase Activity on Chia-Based Networks

Rheological time sweep experiments are frequently used in the characterization of viscoelastic materials to extrapolate information about their mechanical response under oscillatory stimulation [37,38].

**Figure 2:**
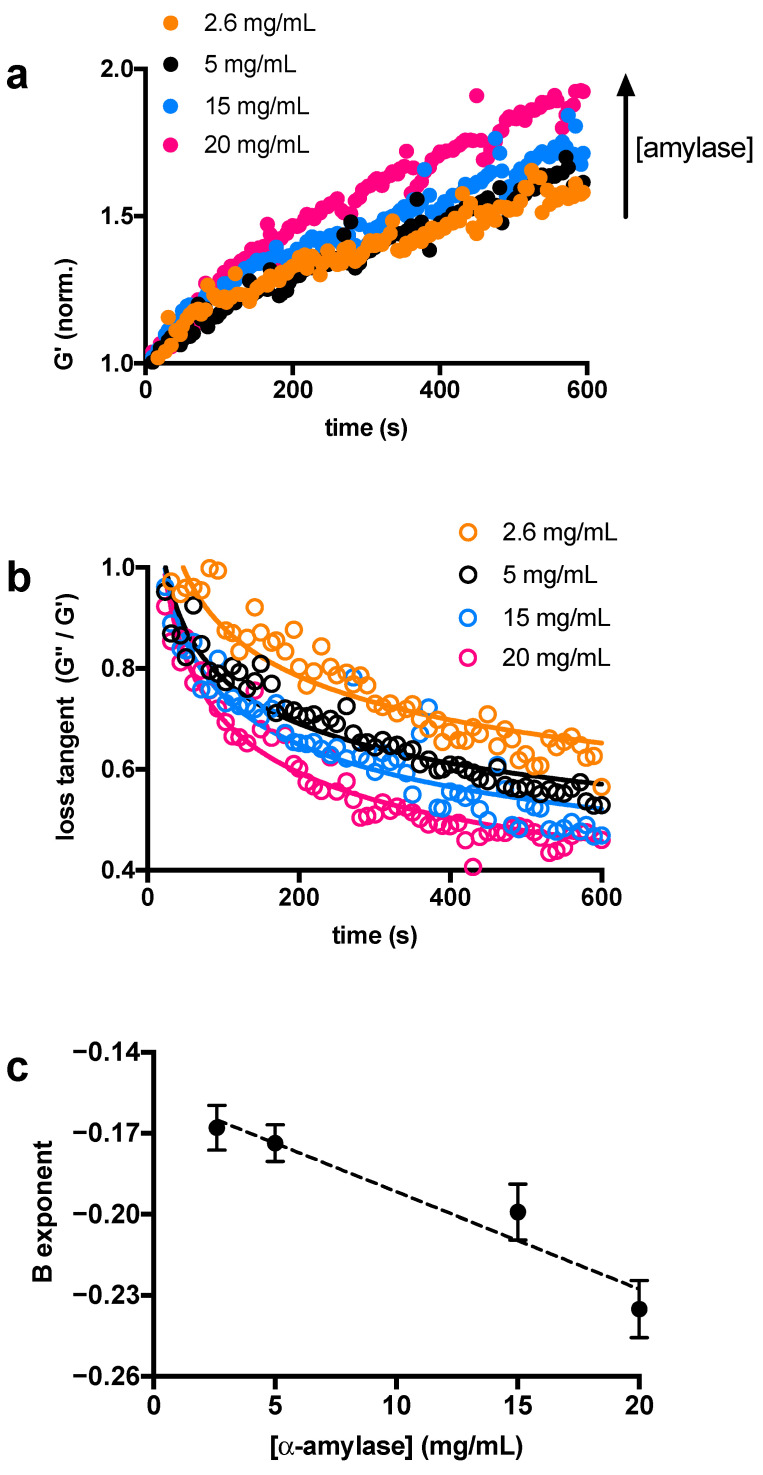
Mechanical changes of chia mucilage suspensions as a function of α-amylase concentration, showing normalized storage modulus G’ (**a**), loss tangent trend over time for each chia-α-amylase mixture (**b**), and exponent *b* () linear dependance on α-amylase concentration (**c**).

With the aim of investigating the role of α-amylase in the structural stability of chia mucilage networks, suspensions of chia were first treated with varying amounts of α-amylase. The experimental set-up involved rapid mixing of a constant amount of chia mucilage (1% *w*/*v*) with different enzyme content (2.6–20 mg/mL) and immediate loading onto the rheometer stage. We decided to use this higher concentration range because in the previous qualitative test, concentrations below 2.6 mg/mL did not seem to have any effect on the solution. Rheological time-sweep tests with constant applied stress and frequency were performed under oscillatory conditions to measure CM structural changes caused by the enzymatic activity. First, the evolution of the CM network was assessed by plotting the normalized storage modulus, G’, as a function of time (Figure 2a).

As apparent from the plot in Figure 2a, the elastic response of CM networks increases with time, whatever the concentration of amylase employed. In particular, the extent of the process depends on the enzyme concentration. This was confirmed by the trend of the loss tangents plotted in the same time scale (Figure 2b). Mathematical treatment of the data presented in Figure 2b revealed that the experimental points scale with a power-law dependence, i.e., *y* = *ax^b^*. Also of interest is the almost linear dependence (*R*^2^ = 0.94) of the exponent *b* as a function of α-amylase concentration (Figure 2c). Table 1 recapitulates the values of *a* and *b* best-fit values. These findings indicate that the networks of CM become more elastic when treated with amylase and that this phenomenon can be controlled by the amount of enzyme used. We speculate that crude fibers play a central role in explaining this behavior since they represent the major (rigid) constituent of the material [22]. Furthermore, IR spectroscopy analysis already demonstrated that this carbohydrate moiety is susceptible to degradation processes [20]. The α-amylase attacks the α-1,4-glucoside bond between the α-D-glucopyranose residue and the second β-D-xylopyranose residue, cleaving the tetrasaccharide (Figure 1b), similar to what happens in the case of starch [26]. Therefore, insoluble and rigid fibers are released in the solution, which are responsible for increasing the elastic modulus of the material, as shown in Figure 1a (close- up).

**Figure 3:**
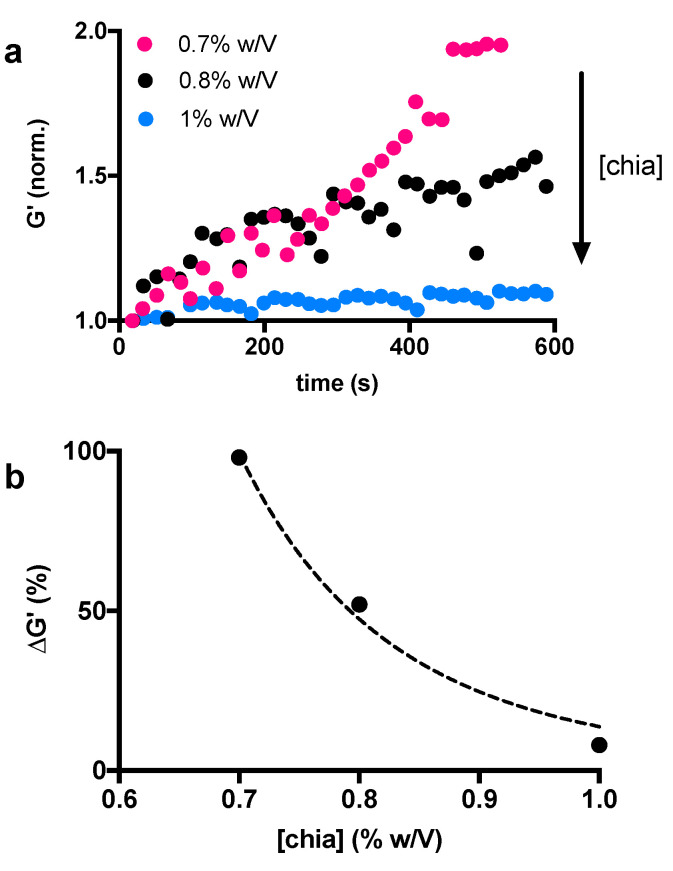
Chia mucilage storage modulus G’ changes over time at varying chia concentrations (0.7%, 0.8% and 1% *w*/*v*) (**a**), and increment of elastic response over time (600 s) as a function of chia concentration (**b**).

The activity of α-amylase can also be influenced by the accessibility of the CM network. Indeed, the diffusion of molecules is influenced by the pore size and the number of junctions within the matrix [39]. In our experimental conditions, the accessibility of the enzyme could be manipulated while varying the concentration of the chia mucilage. It is expected that a higher chia concentration leads to a more packed and less accessible structure. In this case, the diffusion of the α-amylase through the material, as well as the access to the binding sites, is hindered by the hydrogel structure. In order to assess the effect of chia concentrations on the polymer network structure and study its accessibility to α-amylase we decided to vary the chia concentration in the range of 0.7–1% *w*/*v* and keep the *α-*amylase concentration constant. Measurements were performed immediately after the mixing of the two substances. Based on the previous results and on the physiological significance of the enzyme, we selected the 2.6 mg/mL concentration, which corresponds to the average amount of *α-*amylase in saliva [28]. As expected, the results of this second set of time-sweep experiments showed that the elastic response of the CM network increases with time and this trend can be progressively attenuated by increasing the amount of polymer (Figure 3a). This result is made clear by plotting the trend of the maximum elastic response at about 600 s as a function of chia concentration (Figure 3b). These results also show that the structural changes of chia-based hydrogels lead to more elastic networks after amylase treatment, and this phenomenon can be attenuated with increasing amounts of chia. As anticipated above, this can be explained by considering the accessibility of the network. Increasing the chia concentration results in the formation of a denser structure. Therefore, it will be difficult for the enzyme to reach its target, that is the α-1,4-glucoside bond between the α-D-glucopyranose residue and the second β-D-xylopyranose residue. It should be noted that the results shown in Figure 2 and Figure 3 are from two suspensions of the same chia mucilage prepared in two distinct times. Due to the high heterogeneity of chia when dissolved in water, they can therefore not be compared to each other. This explains why the storage modulus of the chia 1% *w*/*v* sample treated with 2.6 mg/mL of *α-*amylase increases by more than 50% in Figure 2a, while the increase in Figure 3a is very small.

These data agree with previous studies performed on starch. It should also be recalled that the differences in the exposure to amylolysis can depend on the various botanical species as well as for chia mucilage on the extraction procedure [31]. It was demonstrated that the entity of the enzyme degradation, and thus the availability of released monomers depends on the viscosity of the hydrogels, which determines the amylase interaction with the specific sides of the polymer. As a whole, our data demonstrate that the presence of α-amylase led to changes in the polymer structure, suggesting the release of insoluble fibers and ensuing increase in the elastic response of chia mucilage suspensions as a possible mechanism.

## 4. Conclusions

Here we studied for the first time the effect of α-amylase, an enzyme commonly found in human saliva, on the structural changes of chia mucilage hydrogels. The process was studied by evaluating visual and mechanical changes during rheological time-sweep tests using two different settings. First, we kept the α-amylase concentration constant while changing the chia mucilage content, then the reverse setting was used. Overall, we showed that the presence of α-amylase leads to significant changes in polymer structure. In particular, the activity of the enzyme leads to more elastic networks as a result of the release of insoluble fibers that constitutively assemble the heterogeneous network of the mucilage. We hypothesize that α-amylase attacks the α-1,4-glucoside bond between the α-D-glucopyranose residue and the second β-D-xylopyranose residue, cleaving the tetrasaccharide and leading to the release of crude fibers of the chia mucilage into the solution, which in turn aggregate and form fibrous structures. The latter help to make the material more elastic under oscillatory mechanical stimulation. Qualitative tests confirmed the presence of these fibrous aggregates, which were visible to the naked eye after 6 h of treatment with 2.6 mg/mL α-amylase, a concentration equivalent to the average amount of α-amylase in saliva. Overall, these data, which are consistent with studies conducted with starch, could be relevant for commercial food processing of small biomolecules.

## Figures and Tables

**Table 1:** Values of *a* and *b* coefficients (*y* = *ax^b^*) for each α-amylase concentration used.

Best-Fit Values	[α-Amylase] (mg/mL)			
	2.6	5	15	20
*a*	1.91 ± 0.09	1.73 ± 0.06	1.87 ± 0.10	2.06 ± 0.12
*b*	−0.168 ± 0.008	−0.174 ± 0.007	−0.199 ± 0.010	−0.235 ± 0.011
